# Heterozygous *PLA2G6* Mutation Leads to Iron Accumulation Within Basal Ganglia and Parkinson's Disease

**DOI:** 10.3389/fneur.2018.00536

**Published:** 2018-07-10

**Authors:** Rosangela Ferese, Simona Scala, Francesca Biagioni, Emiliano Giardina, Stefania Zampatti, Nicola Modugno, Claudio Colonnese, Marianna Storto, Francesco Fornai, Giuseppe Novelli, Stefano Ruggieri, Stefano Gambardella

**Affiliations:** ^1^IRCCS Neuromed, Pozzilli, Italy; ^2^Molecular Genetics Laboratory UILDM, Santa Lucia Foundation, Rome, Italy; ^3^Department of Biomedicine and Prevention, University of Rome Tor Vergata, Rome, Italy; ^4^DAI Neurology and Psichiatry, Department of Neuroradiology, Policlinico Umberto I, Sapienza University of Rome, Rome, Italy; ^5^Department of Translational Research and New Technologies in Medicine and Surgery, University of Pisa, Pisa, Italy

**Keywords:** genetic of Parkinson's disease, incomplete penetrance, PARK14, Parkinson and iron accumulation, L-DOPA responsive

## Abstract

Mutations of PLA2G6 gene are responsible for PARK14, an autosomal recessive L-DOPA responsive dystonia/parkinsonism with early/adult onset. This phenotype possesses an high clinical variability, which consists in the occurrence of cerebral and cerebellar atrophy, iron accumulation in the basal ganglia, and cognitive decline. This report describes a PD patient carrying an heterozygous PLA2G6 mutation, which was identified also in his PD affected sister. This patient is characterized by a L-DOPA responsive typical parkinsonian syndrome without the occurrence of dystonia, a slight cognitive decline, presence of iron accumulation both in neo and paleostriatum while cerebellar atrophy was absent. Clinical and imaging features are compatible with the PARK14 phenotype. Although PARK14 has been previously reported to be inherited as a recessive disorder, clinical and genetic analysis of this proband and his family rise the hypothesis that even heterozygous PLA2G6 mutations may cause PARK14. It remains to be analyzed whether these heterozygous variants may act as dominant mutations, or they merely increase the risk to develop PD by acting within a context of synergistic genetic and/or environmental backgrounds.

## Background

Phospholipase A2-associated neurodegeneration (PLAN), a syndrome of Neurodegeneration with Brain Iron Accumulation (NBIA), is an autosomal recessive neurological disorder caused by mutations in the *PLA2G6* gene (1).

PLAN syndrome encompasses a group of phenotypes like classic infantile neuroaxonal dystrophy (INAD), and atypical neuroaxonal dystrophy of childhood-onset (atypical NAD), characterized by high levels of iron, especially in the globus pallidus (2, 3).

*PLA2G6* gene encodes a calcium-independent group VI phospholipase A2 (iPLA-VI), which is critical in regulating cell membrane homeostasis, mitochondrial function and membrane remodeling (4, 5). Moreover, elevated expression of α-Synuclein (α-Syn) in neuronal mitochondria occurs constantly in PLA2G6-deficiency (6).

Because of its role in the generation of reactive oxygen species and its association with neurodegeneration with brain iron accumulation, an involvement of *PLA2G6* in the pathogenesis of Parkinson's disease (PD) was proposed. In fact, oxidative stress and increased brain iron deposits play a pivotal role in the genesis of PD (7).

In line with this, in 2008 *PLA2G6* gene was shown to be the causative gene underlying PARK14, an autosomal recessive L-DOPA responsive dystonia/parkinsonism with early or adult-onset (8–12). These patients suffer from dystonia, bradykinesia, rigidity, and marked cognitive decline. Subsequently, a range of phenotypes has been reported, from pure Parkinsonism to severe generalized dystonia. While brain MRI shows cerebellar atrophy in almost all well-established PLAN cases, in adult-onset PARK14 little cerebellar atrophy is observed (13–19).

To date, there are about 80 different *PLA2G6* gene abnormalities, and no mutation hot spots have been reported so far. *PLA2G6* genotype-phenotype correlation is described in INAD patients with homozygous null mutations, and in some PARK14 patients, where truncated mutations produce a complex phenotype with cortical and cerebellar atrophy (8, 20–25).

Autosomal recessive inheritance of PARK14 suggests that *PLA2G6* mutations cause the loss of PLA2G6 function and impair the ability of PLA2G6 to protect mitochondria against apoptotic stimuli and exert a neuroprotective effect on dopaminergic cells (4).

In this manuscript, we describe a patient carrying a PLA2G6 mutation which is affected by familial PD, with some clinical and imaging features which are compatible with the PARK14 phenotype. Unexpectedly, this patient carries an heterozygous mutation in the *PLA2G6* gene, despite the evidence provided so far indicates the occurrence of PD only in homozygosis. Strengthening this unique genotype-phenotype correlation, PD was present also in the sister of the proband, who is also a carrier of the heterozygous mutation. Remarkably, in non-carrier sibling PD was not present.

## Clinical presentation

**Figure 1:**
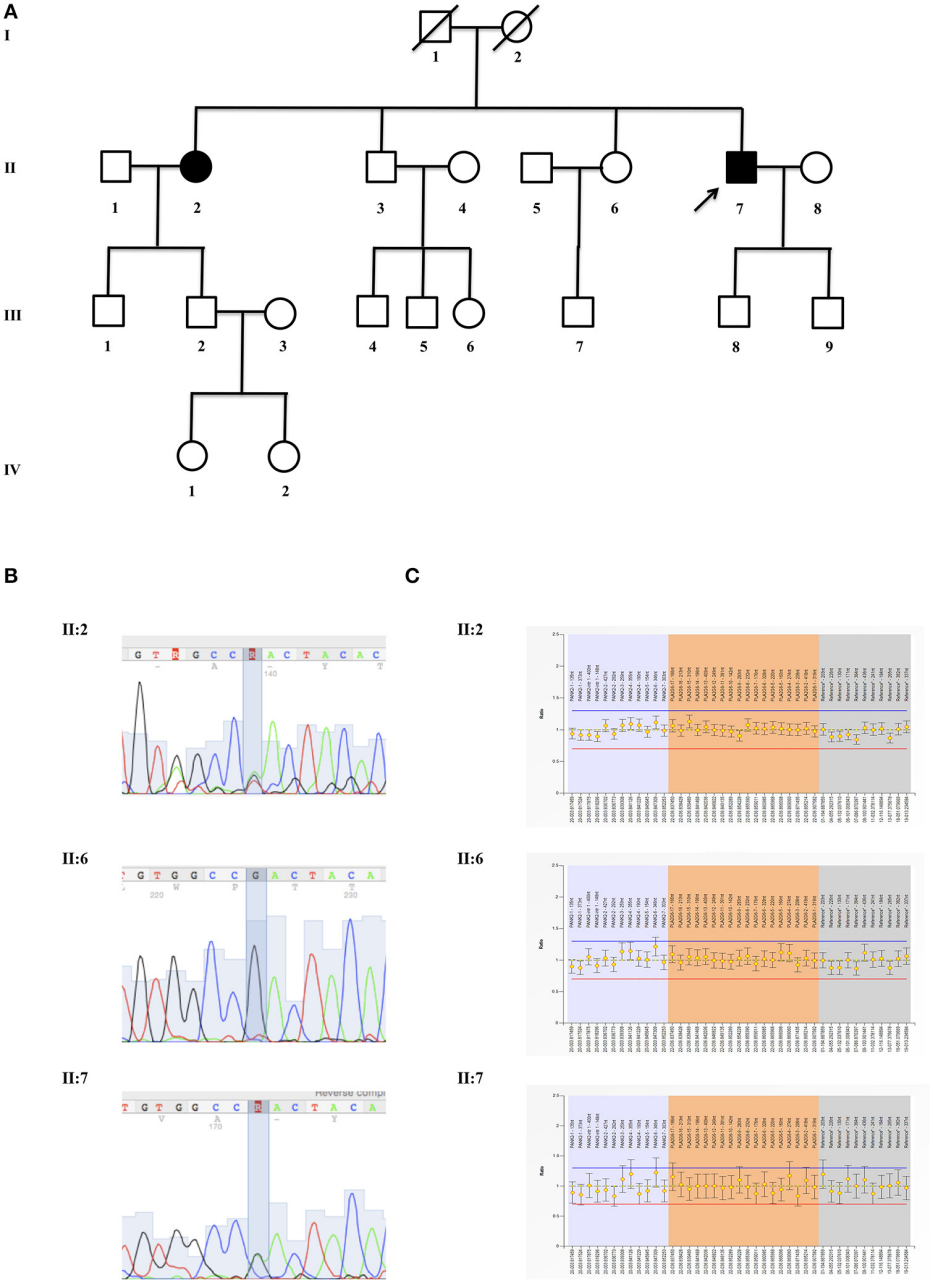
Genetic evaluation. **(A)** Pedigrees of the investigated kindred. Proband (II:7), his affected sister (II:2) and healthy sibling (II:6) are indicated with an arrow; **(B)** mutation analysis of p.Asp31Asn (NP_003551.2), c.91G > A (NM_003560.2:) (rs150024227) in exon 2 of *PLA2G6* gene (OMIM #603604). Sequence analysis is shown for proband (II:7), his affected sister (II:2), and healthy sibling (II:6); **(C)** MLPA analysis of *PLA2G6* gene shows the results for proband (II:7), his affected sister (II:2) and healthy sibling (II:6).

The proband, a 69 years old caucasian man (II:7), was diagnosed with familial PD at the age of 55. Both parents (I:1 and I:2, died, respectively at 85 and 91) and two siblings (II:3, 72 years old and II:6, 71 years old) did not show any neurological sign so far. In contrast, his 74 years old sister (II:2) was recently diagnosed with PD at the age 67 (Figure 1A).

In the medical history of the proband (II:7), no noticeable acute or chronic disorders potentially related to parkinsonism has been reported. In 2004, he presented rigidity of upper and lower left limbs, freezing of gait, and non-motor symptoms (urinary incontinence and reduced olfaction). He was responsive to L-DOPA+benserazide 250+25, which led the UPDRS III score from 17 to 9 during the on- and off-phase respectively. Brain MRI (1,5 Tesla, GE Healthcare), spinal MRI, ECG and evoked potentials were normal at the time of PD diagnosis. The electromyogram (EMG) showed a slight denervation at perineal level.

**Figure 2:**
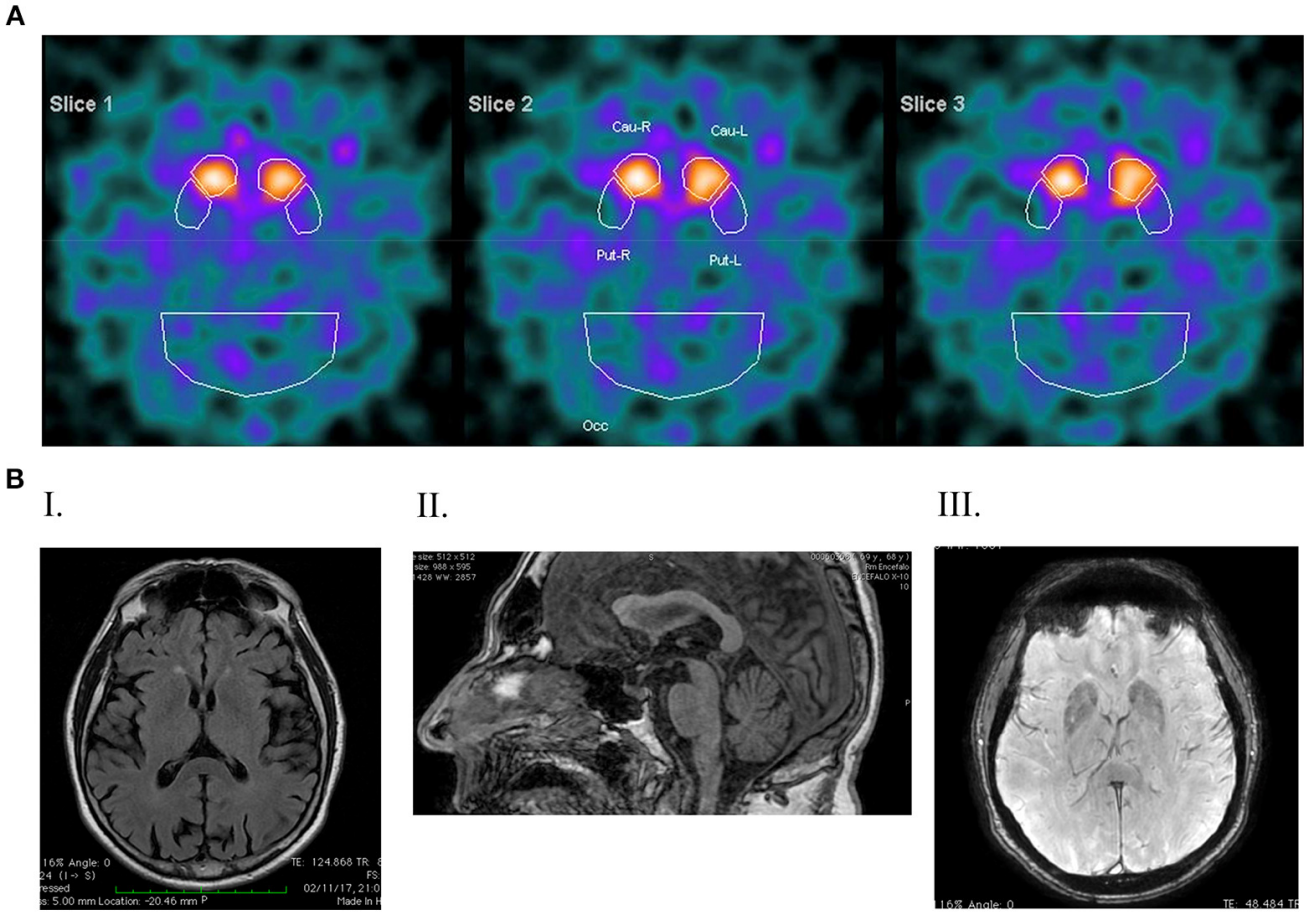
DaTSCAN and Magnetic Resonance Imaging (1,5 Tesla). **(A)** DATSCAN image showing a reduced tracer uptake in both striatal regions; **(B)** magnetic resonance imaging. **(I)** T2 FLAIR shows post-central atrophy and bilateral temporo-insular atrophy; **(II)** SWAN shows a reduced thickness of the mesencephalic tagmen; **(III)** T1 FSPGR shows signs of iron deposition in the globus pallidum and in both putamen.

Three years later a DAT-Scan was carried out, suggesting a marked asymmetric reduction of dopamine (DA) uptake sites within the right basal ganglia (Figure 2A). Five years later, in 2012, DAT-Scan consistently indicated nigro-striatal denervation. Neuropsychological assessment (MMSE) was reported within the normal range.

In 2014 brain MRI showed enlargement of sub-aracnoid spaces bilaterally, surrounding frontal lobes. ECG reported abnormal heart rate recovery. The UPDRS-III registered a deterioration (off = 42, on = 12), and electroneurography was consistent with mild axonal sensory polyneuropathy. There was still no cognitive deterioration at MMSE, while neither mood nor obsessive disorders were reported.

In 2017, the UPDRS-III in the on phase was 20 and a gait disturbance appeared along with involuntary oro-facial dyskinesia. Brain MRI revealed post-central atrophy and bilateral temporo-insular atrophy (Figure 2B-I), along with a reduced thickness of the mesencephalic tegmentum (Figure 2B-II). There was no cerebellar atrophy, while in the basal ganglia brain iron accumulation was remarkable involving the head of the caudate nucleus, putamen and globus pallidus (Figure 2B-III). Spinal MRI, electrocardiogram, electromyogram and electroneuronography were unchanged compared with 2014. At this time a slight cognitive deterioration appeared (MMSE 23/30).

A written informed consent for genetic analysis was obtained, and clinical exome sequencing considered 4800 human genes including 17 genes related to Parkinson disease (PARK1:*SNCA*; PARK2:*PRKN*; PARK3:*SPR*; PARK5:*UCHL1*; PARK6:*PINK1*; PARK7:*DJ1*; PARK8: *LRRK2*; PARK9:*ATP13A2*; PARK10:*ELAVL4*; PARK11:*GIGYF2*; PARK12:*TAF1*; PARK13:*HTRA2*; PARK14:*PLA2G6*; PARK15:*FBXO7*; PARK16:*ADORA1*; PARK17:*VPS35*; PARK18:*EIF4GI*) (TruSight One Sequencing Panels, Illumina) was performed. Sequence analysis identified the mutation p.Asp31Asn (NP_003551.2), c.91G>A (NM_003560.2:) (rs150024227) in *PLA2G6* gene (OMIM #603604), confirmed by Sanger sequencing (Figure 1B). No mutations in other PD-related genes were identified. Multiple-ligation probe amplification (MLPA) (SALSA MLPA kit P120-B2, MRC-Holland) ruled out the presence of duplication or deletion as second mutation (Figure 1C). After a genetic counseling, the presence of p.Asp31Asn was evaluated in II:2 (PD affected sister just diagnosed) and II:6 (healthy sister). The same p.Asp31Asn variant was confirmed in II:2, while it was not present in asymptomatic II:6. Unfortunately, it was impossible to evaluate this variant in the parents (I:1 and I:2) and in one healthy brother (II:3).

## Discussion

This manuscript describes a patient with familial PD, who carries a p.Asp31Asn variant in *PLA2G6*, the causative gene of PARK14. This PD phenotype is characterized by parkinsonism with dystonia. Clinical variability consists in the occurrence of cerebral and cerebellar atrophy, iron accumulation in the basal ganglia, age at onset and cognitive decline.

This patient is characterized by a L-DOPA responsive typical parkinsonian syndrome without the occurrence of dystonia even at 15 years after PD diagnosis. At longer time interval, a slight cognitive decline appeared. MRI showed the presence of iron accumulation both in neo and paleostriatum while cerebellar atrophy was absent. No data about disease progression are available so far for the newly diagnosed sister (II:2) who had a delayed disease onset compared with the patient reported (67 and 55, respectively).

PARK14 was been described as a recessive disease, where two mutations (homozygous or compound heterozygous) in *PLA2G6* gene were always present. The patient described here is uniques since he carries a single mutation affecting the *PLA2G6*. This variant segregated with PD in II:2 (PD affected sister just diagnosed), while it was not present II:6 (healthy sister).

These findings pose the question about the role of heterozygosity in genes related with PARK 14 and, in general, autosomal recessive parkinsonism. In fact, specific allelic variants in the PARK14 locus may lead to PD with an autosomal dominant inheritance (being still unknown the penetrance). In keeping with this, a recent report describes an heterozygous mutation in PARK14 causing PD in a 69 years old patient (23). This patient was reported to carry all typical features of L-DOPA responsive PD associated with dystonia, but no evidence of iron deposition. It could be speculated that only a few mutations in PLP2G6 could act as dominant, producing a specific kind of parkinsonism as witnessed by the phenotype described here compared with other PARK14 patients with two *PLA2G6* variants. Further analysis are required to firmly establish whether specific heterozygous mutations of PARK14 may lead to PD.

Another potential explanation is that heterozygote mutations in genes related with autosomal recessive parkinsonism lead to increased risk of PD. In these scenario, the present data contribute to the working hypothesis that *PLA2G6* heterozygote mutations may represent a risk factor for PD, as reported for some *PLA2G6* mutations in idiopathic PD patients (25). This is supported by the frequency of this variant in different populations (ExAC database: minor allele frequency = 0.0004), and by the presence of single variants reported in sporadic PD (14). It is fascinating that recent studies report PLA2G6 immunostaining in the core of brainstem-type Lewy bodies from PARK14 PD patients and, most remarkably, in idiopathic PD (26, 27). This witnesses for a role of PLA2G6 in the physiopathology of PD thus remarking the potential role of environmental interactions as well as genetic background.

However, it is still debated whether heterozygous carriers of mutations of genes related with autosomal recessive parkinsonism own an increased risk to develop PD (28). Although more than 50% of patients with mutations in parkin or PINK1 have only a single heterozygous mutation, the few large studies that assessed the frequency of heterozygotes remain controversial (29, 30). In fact, while a retrospective analysis of the occurrence of PINK1 heterozygous rare variants in PD could not detect significant differences compared with controls, another study reported that heterozygous CNVs (Copy Number Variations) in PARK2 gene could confer an higher risk to develop PD (28, 30).

Therefore, although the pathogenic significance of these variants remains uncertain, they may represent a risk factor to develop PD, supporting the hypothesis that haplo-insufficiency is a more likely explanation than compound heterozygosity through unidentified recessive mutations.

## Conclusions

In this manuscript, we describe a patient carrying a PLA2G6 mutation which is affected by familial PD, with some clinical and imaging features which are compatible with the PARK14 phenotype. This patient is characterized by a L-DOPA responsive typical parkinsonian syndrome without the occurrence of dystonia, a slight cognitive decline, presence of iron accumulation both in neo and paleostriatum while cerebellar atrophy was absent.

Although PARK14 has been reported so far as a recessive disease, clinical and genetic analysis of the proband and his family rise the hypothesis of a potential role of some heterozygous *PLA2G6* mutations in causing PARK14. It remains to be analyzed in detail whether these variants act really as dominant PD-inducing mutations, or they play a role as risk factors acting within synergistic genetic and/or environment backgrounds. Deciphering the role of heterozygosity in parkinsonism is important for the development of guidelines, for genetic testing and counseling, and for the understanding of late-onset Parkinson's disease.

## Ethics statement

Approved by the IRCCS Neuromed INM Ethics Committee. Written informed consent was obtained from the participant for the publication of this case report. Protocol ID:CGM-01 Clinical Trials ID:NCT03084224

## Author contributions

SG, RF, and FB planned and designed the study. RF and SS test execution, acquired and analyzed the data. RF and SG drafting of manuscript. SZ, NM, CC, EG, and MS pathology and clinical data. FF, GN, and SR critical revisions. All authors approved submission of the manuscript.

### Conflict of interest statement

The authors declare that the research was conducted in the absence of any commercial or financial relationships that could be construed as a potential conflict of interest. The handling Editor declared a shared affiliation, though no other collaboration, with several of the authors EG and GN.
